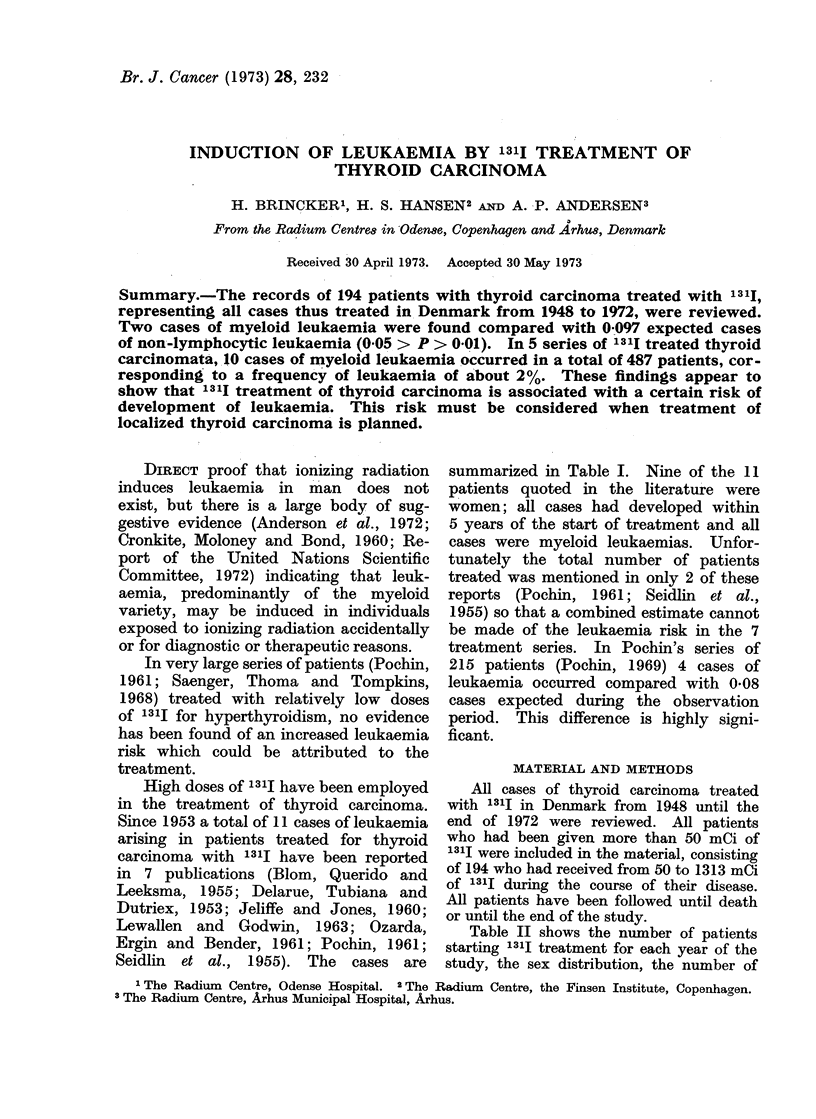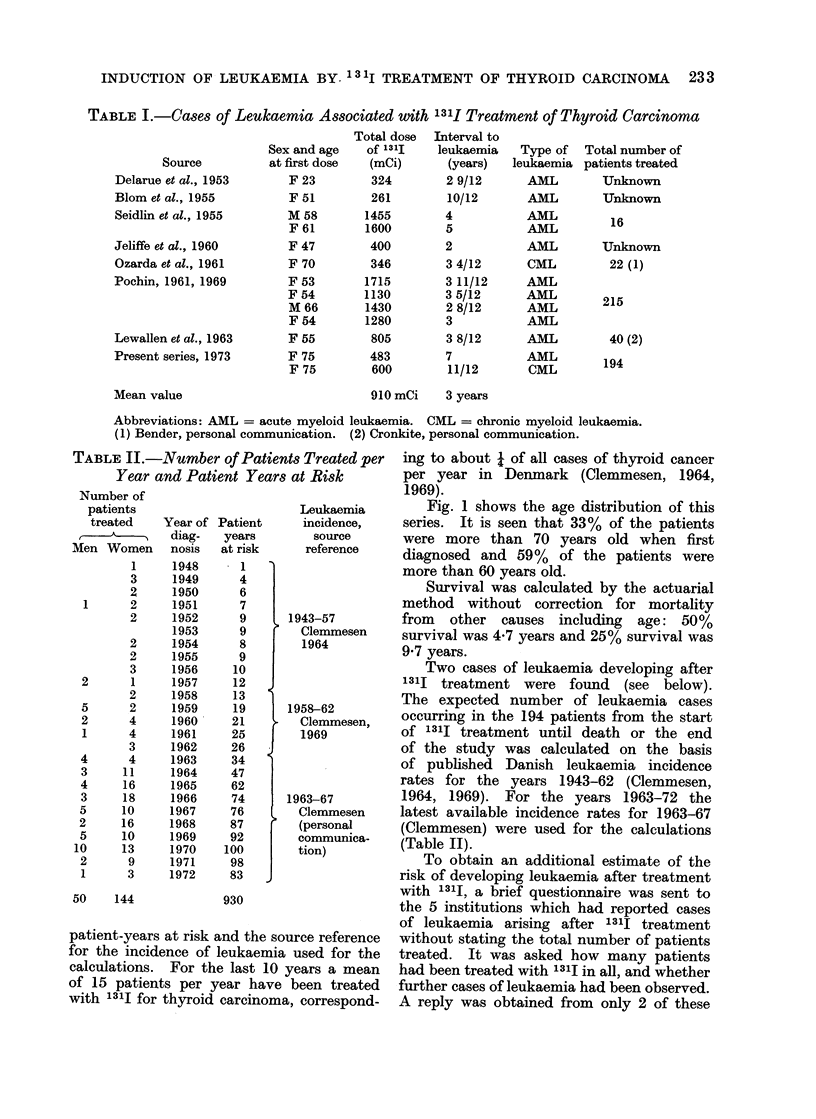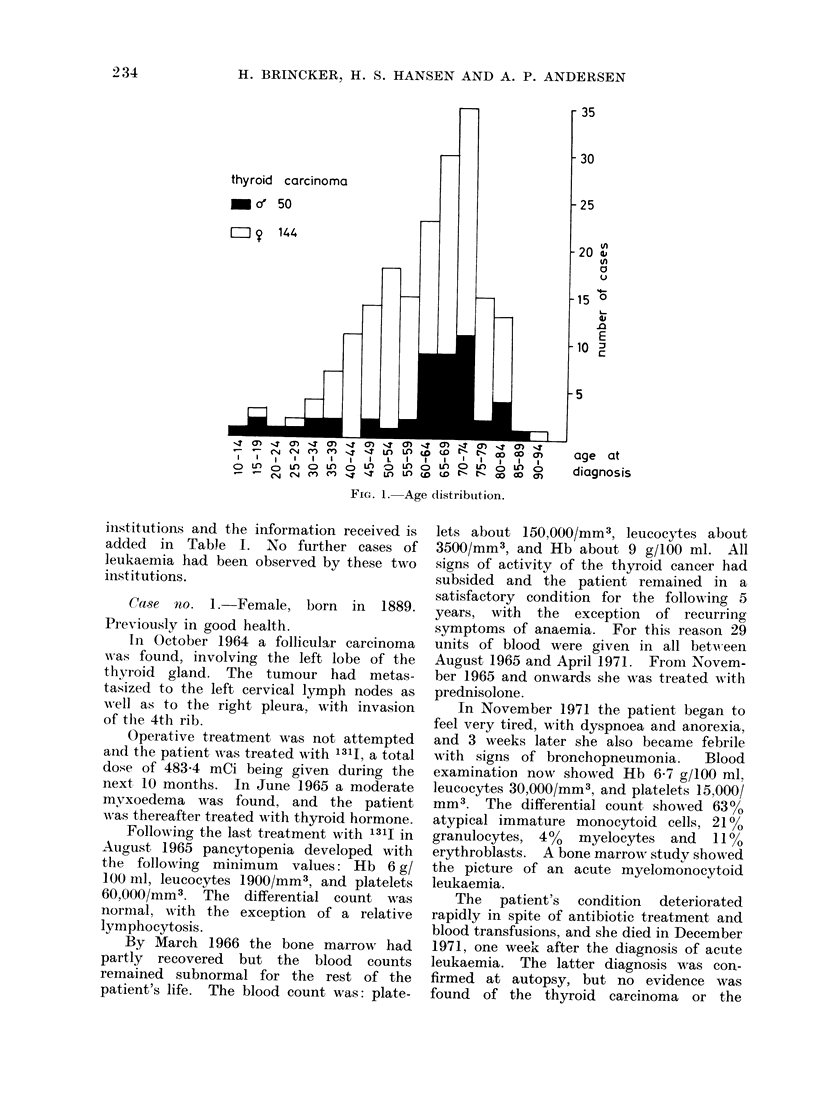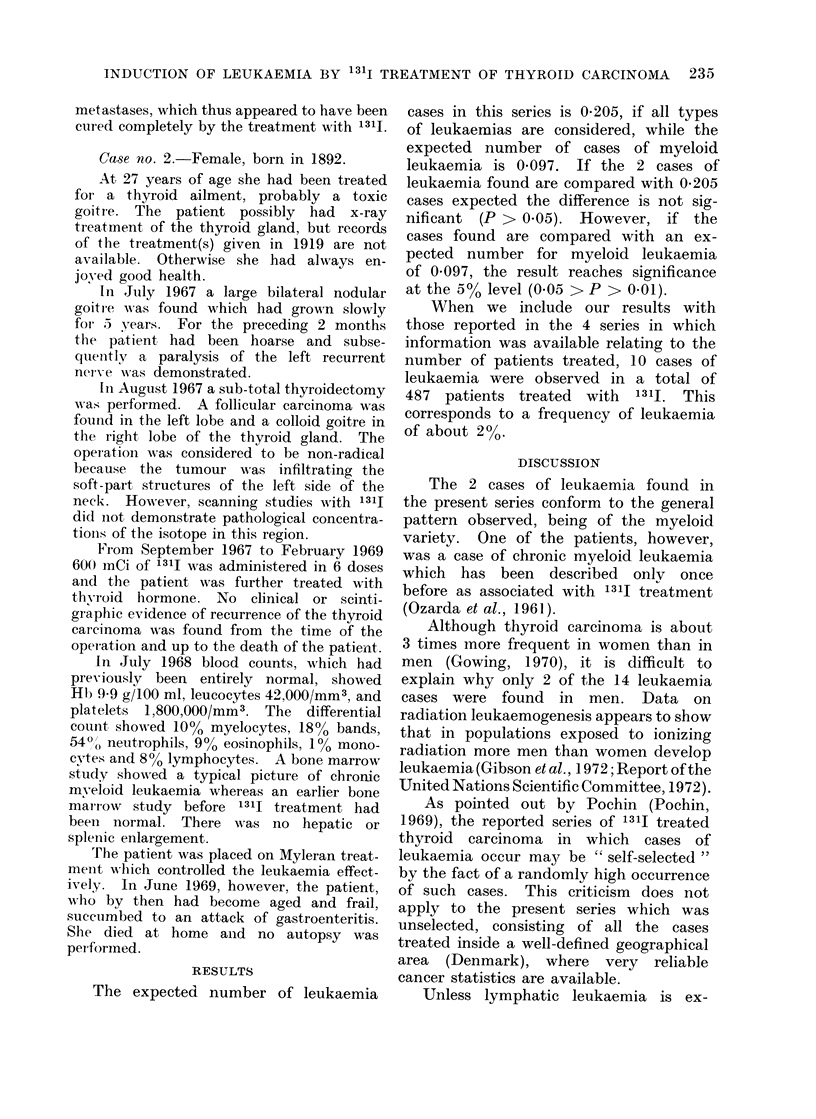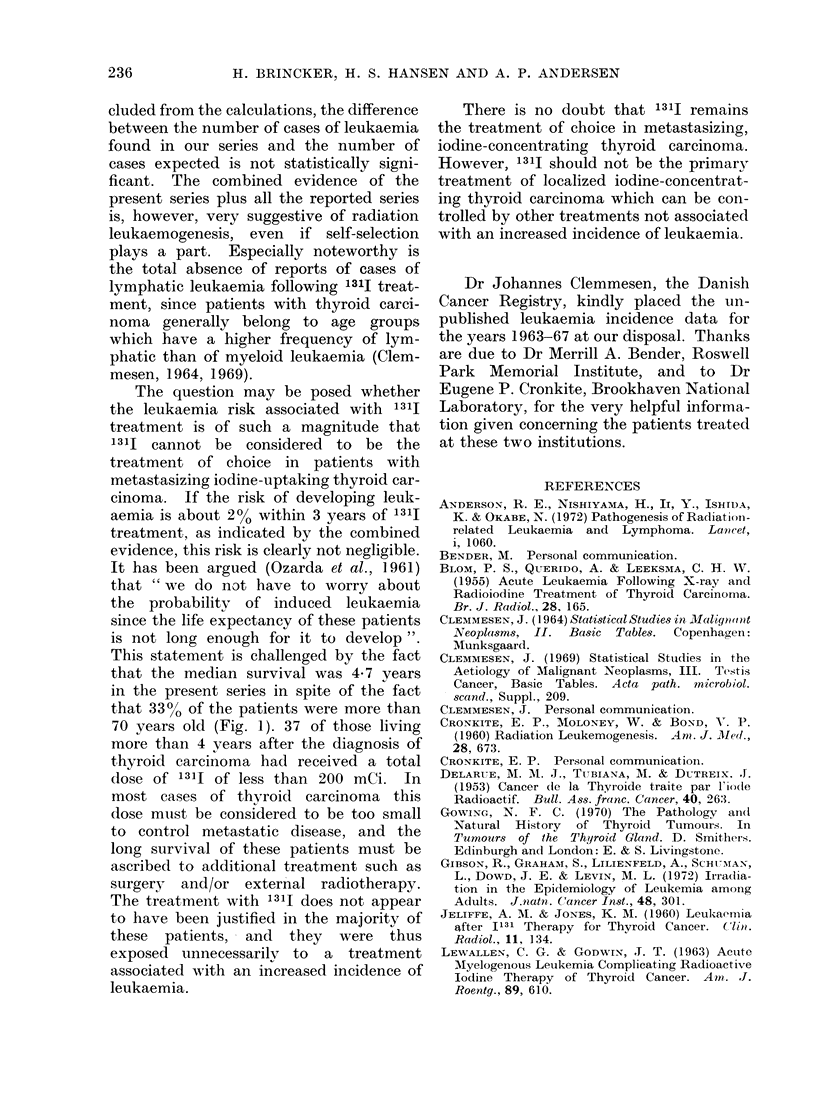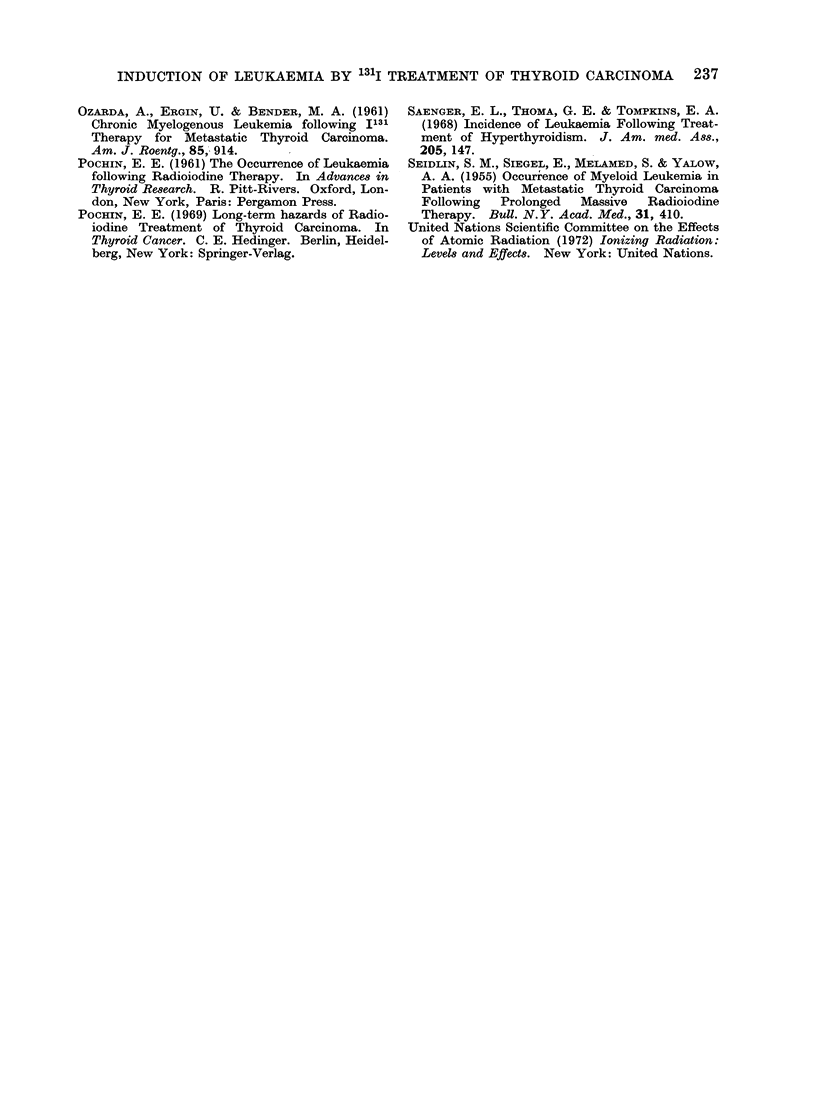# Induction of Leukaemia by ^131^I Treatment of Thyroid Carcinoma

**DOI:** 10.1038/bjc.1973.142

**Published:** 1973-09

**Authors:** H. Brincker, H. S. Hansen, A. P. Andersen

## Abstract

The records of 194 patients with thyroid carcinoma treated with ^131^I, representing all cases thus treated in Denmark from 1948 to 1972, were reviewed. Two cases of myeloid leukaemia were found compared with 0·097 expected cases of non-lymphocytic leukaemia (0·05 > *P* > 0·01). In 5 series of ^131^I treated thyroid carcinomata, 10 cases of myeloid leukaemia occurred in a total of 487 patients, corresponding to a frequency of leukaemia of about 2%. These findings appear to show that ^131^I treatment of thyroid carcinoma is associated with a certain risk of development of leukaemia. This risk must be considered when treatment of localized thyroid carcinoma is planned.


Br. J. Cancer (1973) 28, 232

### INDUCTION OF LEUKAEMIA BY $^{131}I$ TREATMENT OF THYROID CARCINOMA

H. BRINCKER[1], H. S. HANSEN[2] AND A. P. ANDERSEN[3]

*From the Radium Centres in Odense, Copenhagen and Århus, Denmark*

Received 30 April 1973. Accepted 30 May 1973

**Summary.—The records of 194 patients with thyroid carcinoma treated with $^{131}I$, representing all cases thus treated in Denmark from 1948 to 1972, were reviewed. Two cases of myeloid leukaemia were found compared with 0·097 expected cases of non-lymphocytic leukaemia ($0{\cdot}05 > P > 0{\cdot}01$). In 5 series of $^{131}I$ treated thyroid carcinomata, 10 cases of myeloid leukaemia occurred in a total of 487 patients, corresponding to a frequency of leukaemia of about 2%. These findings appear to show that $^{131}I$ treatment of thyroid carcinoma is associated with a certain risk of development of leukaemia. This risk must be considered when treatment of localized thyroid carcinoma is planned.**

DIRECT proof that ionizing radiation induces leukaemia in man does not exist, but there is a large body of suggestive evidence (Anderson *et al.*, 1972; Cronkite, Moloney and Bond, 1960; Report of the United Nations Scientific Committee, 1972) indicating that leukaemia, predominantly of the myeloid variety, may be induced in individuals exposed to ionizing radiation accidentally or for diagnostic or therapeutic reasons.

In very large series of patients (Pochin, 1961; Saenger, Thoma and Tompkins, 1968) treated with relatively low doses of $^{131}I$ for hyperthyroidism, no evidence has been found of an increased leukaemia risk which could be attributed to the treatment.

High doses of $^{131}I$ have been employed in the treatment of thyroid carcinoma. Since 1953 a total of 11 cases of leukaemia arising in patients treated for thyroid carcinoma with $^{131}I$ have been reported in 7 publications (Blom, Querido and Leeksma, 1955; Delarue, Tubiana and Dutriex, 1953; Jeliffe and Jones, 1960; Lewallen and Godwin, 1963; Ozarda, Ergin and Bender, 1961; Pochin, 1961; Seidlin *et al.*, 1955). The cases are summarized in Table I. Nine of the 11 patients quoted in the literature were women; all cases had developed within 5 years of the start of treatment and all cases were myeloid leukaemias. Unfortunately the total number of patients treated was mentioned in only 2 of these reports (Pochin, 1961; Seidlin *et al.*, 1955) so that a combined estimate cannot be made of the leukaemia risk in the 7 treatment series. In Pochin's series of 215 patients (Pochin, 1969) 4 cases of leukaemia occurred compared with 0·08 cases expected during the observation period. This difference is highly significant.

### MATERIAL AND METHODS

All cases of thyroid carcinoma treated with $^{131}I$ in Denmark from 1948 until the end of 1972 were reviewed. All patients who had been given more than 50 mCi of $^{131}I$ were included in the material, consisting of 194 who had received from 50 to 1313 mCi of $^{131}I$ during the course of their disease. All patients have been followed until death or until the end of the study.

Table II shows the number of patients starting $^{131}I$ treatment for each year of the study, the sex distribution, the number of

[1] The Radium Centre, Odense Hospital. [2] The Radium Centre, the Finsen Institute, Copenhagen. [3] The Radium Centre, Århus Municipal Hospital, Århus.

TABLE I.—*Cases of Leukaemia Associated with $^{131}$I Treatment of Thyroid Carcinoma*

| Source | Sex and age at first dose | Total dose of $^{131}$I (mCi) | Interval to leukaemia (years) | Type of leukaemia | Total number of patients treated |
|---|---|---|---|---|---|
| Delarue *et al.*, 1953 | F 23 | 324 | 2 9/12 | AML | Unknown |
| Blom *et al.*, 1955 | F 51 | 261 | 10/12 | AML | Unknown |
| Seidlin *et al.*, 1955 | M 58 | 1455 | 4 | AML | 16 |
| | F 61 | 1600 | 5 | AML | |
| Jeliffe *et al.*, 1960 | F 47 | 400 | 2 | AML | Unknown |
| Ozarda *et al.*, 1961 | F 70 | 346 | 3 4/12 | CML | 22 (1) |
| Pochin, 1961, 1969 | F 53 | 1715 | 3 11/12 | AML | 215 |
| | F 54 | 1130 | 3 5/12 | AML | |
| | M 66 | 1430 | 2 8/12 | AML | |
| | F 54 | 1280 | 3 | AML | |
| Lewallen *et al.*, 1963 | F 55 | 805 | 3 8/12 | AML | 40 (2) |
| Present series, 1973 | F 75 | 483 | 7 | AML | 194 |
| | F 75 | 600 | 11/12 | CML | |
| Mean value | | 910 mCi | 3 years | | |

Abbreviations: AML = acute myeloid leukaemia. CML = chronic myeloid leukaemia.
(1) Bender, personal communication. (2) Cronkite, personal communication.

TABLE II.—*Number of Patients Treated per Year and Patient Years at Risk*

| Number of patients treated: Men | Women | Year of diagnosis | Patient years at risk | Leukaemia incidence, source reference |
|---|---|---|---|---|
| | 1 | 1948 | 1 | 1943–57 Clemmesen 1964 |
| | 3 | 1949 | 4 | |
| | 2 | 1950 | 6 | |
| 1 | 2 | 1951 | 7 | |
| | 2 | 1952 | 9 | |
| | | 1953 | 9 | |
| | 2 | 1954 | 8 | |
| | 2 | 1955 | 9 | |
| | 3 | 1956 | 10 | |
| 2 | 1 | 1957 | 12 | |
| | 2 | 1958 | 13 | 1958–62 Clemmesen, 1969 |
| 5 | 2 | 1959 | 19 | |
| 2 | 4 | 1960 | 21 | |
| 1 | 4 | 1961 | 25 | |
| | 3 | 1962 | 26 | |
| 4 | 4 | 1963 | 34 | 1963–67 Clemmesen (personal communication) |
| 3 | 11 | 1964 | 47 | |
| 4 | 16 | 1965 | 62 | |
| 3 | 18 | 1966 | 74 | |
| 5 | 10 | 1967 | 76 | |
| 2 | 16 | 1968 | 87 | |
| 5 | 10 | 1969 | 92 | |
| 10 | 13 | 1970 | 100 | |
| 2 | 9 | 1971 | 98 | |
| 1 | 3 | 1972 | 83 | |
| 50 | 144 | | 930 | |

patient-years at risk and the source reference for the incidence of leukaemia used for the calculations. For the last 10 years a mean of 15 patients per year have been treated with $^{131}$I for thyroid carcinoma, corresponding to about $\frac{1}{4}$ of all cases of thyroid cancer per year in Denmark (Clemmesen, 1964, 1969).

Fig. 1 shows the age distribution of this series. It is seen that 33% of the patients were more than 70 years old when first diagnosed and 59% of the patients were more than 60 years old.

Survival was calculated by the actuarial method without correction for mortality from other causes including age: 50% survival was 4·7 years and 25% survival was 9·7 years.

Two cases of leukaemia developing after $^{131}$I treatment were found (see below). The expected number of leukaemia cases occurring in the 194 patients from the start of $^{131}$I treatment until death or the end of the study was calculated on the basis of published Danish leukaemia incidence rates for the years 1943–62 (Clemmesen, 1964, 1969). For the years 1963–72 the latest available incidence rates for 1963–67 (Clemmesen) were used for the calculations (Table II).

To obtain an additional estimate of the risk of developing leukaemia after treatment with $^{131}$I, a brief questionnaire was sent to the 5 institutions which had reported cases of leukaemia arising after $^{131}$I treatment without stating the total number of patients treated. It was asked how many patients had been treated with $^{131}$I in all, and whether further cases of leukaemia had been observed. A reply was obtained from only 2 of these

FIG. 1.—Age distribution.

institutions and the information received is added in Table I. No further cases of leukaemia had been observed by these two institutions.

*Case no.* 1.—Female, born in 1889. Previously in good health.

In October 1964 a follicular carcinoma was found, involving the left lobe of the thyroid gland. The tumour had metastasized to the left cervical lymph nodes as well as to the right pleura, with invasion of the 4th rib.

Operative treatment was not attempted and the patient was treated with $^{131}I$, a total dose of 483·4 mCi being given during the next 10 months. In June 1965 a moderate myxoedema was found, and the patient was thereafter treated with thyroid hormone.

Following the last treatment with $^{131}I$ in August 1965 pancytopenia developed with the following minimum values: Hb 6 g/100 ml, leucocytes 1900/mm$^3$, and platelets 60,000/mm$^3$. The differential count was normal, with the exception of a relative lymphocytosis.

By March 1966 the bone marrow had partly recovered but the blood counts remained subnormal for the rest of the patient's life. The blood count was: platelets about 150,000/mm$^3$, leucocytes about 3500/mm$^3$, and Hb about 9 g/100 ml. All signs of activity of the thyroid cancer had subsided and the patient remained in a satisfactory condition for the following 5 years, with the exception of recurring symptoms of anaemia. For this reason 29 units of blood were given in all between August 1965 and April 1971. From November 1965 and onwards she was treated with prednisolone.

In November 1971 the patient began to feel very tired, with dyspnoea and anorexia, and 3 weeks later she also became febrile with signs of bronchopneumonia. Blood examination now showed Hb 6·7 g/100 ml, leucocytes 30,000/mm$^3$, and platelets 15,000/mm$^3$. The differential count showed 63% atypical immature monocytoid cells, 21% granulocytes, 4% myelocytes and 11% erythroblasts. A bone marrow study showed the picture of an acute myelomonocytoid leukaemia.

The patient's condition deteriorated rapidly in spite of antibiotic treatment and blood transfusions, and she died in December 1971, one week after the diagnosis of acute leukaemia. The latter diagnosis was confirmed at autopsy, but no evidence was found of the thyroid carcinoma or the metastases, which thus appeared to have been cured completely by the treatment with $^{131}$I.

*Case no.* 2.—Female, born in 1892.

At 27 years of age she had been treated for a thyroid ailment, probably a toxic goitre. The patient possibly had x-ray treatment of the thyroid gland, but records of the treatment(s) given in 1919 are not available. Otherwise she had always enjoyed good health.

In July 1967 a large bilateral nodular goitre was found which had grown slowly for 5 years. For the preceding 2 months the patient had been hoarse and subsequently a paralysis of the left recurrent nerve was demonstrated.

In August 1967 a sub-total thyroidectomy was performed. A follicular carcinoma was found in the left lobe and a colloid goitre in the right lobe of the thyroid gland. The operation was considered to be non-radical because the tumour was infiltrating the soft-part structures of the left side of the neck. However, scanning studies with $^{131}$I did not demonstrate pathological concentrations of the isotope in this region.

From September 1967 to February 1969 600 mCi of $^{131}$I was administered in 6 doses and the patient was further treated with thyroid hormone. No clinical or scintigraphic evidence of recurrence of the thyroid carcinoma was found from the time of the operation and up to the death of the patient.

In July 1968 blood counts, which had previously been entirely normal, showed Hb 9·9 g/100 ml, leucocytes 42,000/mm$^3$, and platelets 1,800,000/mm$^3$. The differential count showed 10% myelocytes, 18% bands, 54% neutrophils, 9% eosinophils, 1% monocytes and 8% lymphocytes. A bone marrow study showed a typical picture of chronic myeloid leukaemia whereas an earlier bone marrow study before $^{131}$I treatment had been normal. There was no hepatic or splenic enlargement.

The patient was placed on Myleran treatment which controlled the leukaemia effectively. In June 1969, however, the patient, who by then had become aged and frail, succumbed to an attack of gastroenteritis. She died at home and no autopsy was performed.

### RESULTS

The expected number of leukaemia cases in this series is 0·205, if all types of leukaemias are considered, while the expected number of cases of myeloid leukaemia is 0·097. If the 2 cases of leukaemia found are compared with 0·205 cases expected the difference is not significant ($P > 0{\cdot}05$). However, if the cases found are compared with an expected number for myeloid leukaemia of 0·097, the result reaches significance at the 5% level ($0{\cdot}05 > P > 0{\cdot}01$).

When we include our results with those reported in the 4 series in which information was available relating to the number of patients treated, 10 cases of leukaemia were observed in a total of 487 patients treated with $^{131}$I. This corresponds to a frequency of leukaemia of about 2%.

### DISCUSSION

The 2 cases of leukaemia found in the present series conform to the general pattern observed, being of the myeloid variety. One of the patients, however, was a case of chronic myeloid leukaemia which has been described only once before as associated with $^{131}$I treatment (Ozarda *et al.*, 1961).

Although thyroid carcinoma is about 3 times more frequent in women than in men (Gowing, 1970), it is difficult to explain why only 2 of the 14 leukaemia cases were found in men. Data on radiation leukaemogenesis appears to show that in populations exposed to ionizing radiation more men than women develop leukaemia (Gibson *et al.*, 1972; Report of the United Nations Scientific Committee, 1972).

As pointed out by Pochin (Pochin, 1969), the reported series of $^{131}$I treated thyroid carcinoma in which cases of leukaemia occur may be " self-selected " by the fact of a randomly high occurrence of such cases. This criticism does not apply to the present series which was unselected, consisting of all the cases treated inside a well-defined geographical area (Denmark), where very reliable cancer statistics are available.

Unless lymphatic leukaemia is excluded from the calculations, the difference between the number of cases of leukaemia found in our series and the number of cases expected is not statistically significant. The combined evidence of the present series plus all the reported series is, however, very suggestive of radiation leukaemogenesis, even if self-selection plays a part. Especially noteworthy is the total absence of reports of cases of lymphatic leukaemia following $^{131}I$ treatment, since patients with thyroid carcinoma generally belong to age groups which have a higher frequency of lymphatic than of myeloid leukaemia (Clemmesen, 1964, 1969).

The question may be posed whether the leukaemia risk associated with $^{131}I$ treatment is of such a magnitude that $^{131}I$ cannot be considered to be the treatment of choice in patients with metastasizing iodine-uptaking thyroid carcinoma. If the risk of developing leukaemia is about 2% within 3 years of $^{131}I$ treatment, as indicated by the combined evidence, this risk is clearly not negligible. It has been argued (Ozarda *et al.*, 1961) that " we do not have to worry about the probability of induced leukaemia since the life expectancy of these patients is not long enough for it to develop ". This statement is challenged by the fact that the median survival was 4·7 years in the present series in spite of the fact that 33% of the patients were more than 70 years old (Fig. 1). 37 of those living more than 4 years after the diagnosis of thyroid carcinoma had received a total dose of $^{131}I$ of less than 200 mCi. In most cases of thyroid carcinoma this dose must be considered to be too small to control metastatic disease, and the long survival of these patients must be ascribed to additional treatment such as surgery and/or external radiotherapy. The treatment with $^{131}I$ does not appear to have been justified in the majority of these patients, and they were thus exposed unnecessarily to a treatment associated with an increased incidence of leukaemia.

There is no doubt that $^{131}I$ remains the treatment of choice in metastasizing, iodine-concentrating thyroid carcinoma. However, $^{131}I$ should not be the primary treatment of localized iodine-concentrating thyroid carcinoma which can be controlled by other treatments not associated with an increased incidence of leukaemia.

Dr Johannes Clemmesen, the Danish Cancer Registry, kindly placed the unpublished leukaemia incidence data for the years 1963–67 at our disposal. Thanks are due to Dr Merrill A. Bender, Roswell Park Memorial Institute, and to Dr Eugene P. Cronkite, Brookhaven National Laboratory, for the very helpful information given concerning the patients treated at these two institutions.

### REFERENCES

ANDERSON, R. E., NISHIYAMA, H., II, Y., ISHIDA, K. & OKABE, N. (1972) Pathogenesis of Radiation-related Leukaemia and Lymphoma. *Lancet*, i, 1060.

BENDER, M. Personal communication.

BLOM, P. S., QUERIDO, A. & LEEKSMA, C. H. W. (1955) Acute Leukaemia Following X-ray and Radioiodine Treatment of Thyroid Carcinoma. *Br. J. Radiol.*, **28**, 165.

CLEMMESEN, J. (1964) *Statistical Studies in Malignant Neoplasms, II. Basic Tables.* Copenhagen: Munksgaard.

CLEMMESEN, J. (1969) Statistical Studies in the Aetiology of Malignant Neoplasms, III. Testis Cancer, Basic Tables. *Acta path. microbiol. scand.*, Suppl., 209.

CLEMMESEN, J. Personal communication.

CRONKITE, E. P., MOLONEY, W. & BOND, V. P. (1960) Radiation Leukemogenesis. *Am. J. Med.*, **28**, 673.

CRONKITE, E. P. Personal communication.

DELARUE, M. M. J., TUBIANA, M. & DUTREIX, J. (1953) Cancer de la Thyroide traite par l'iode Radioactif. *Bull. Ass. franc. Cancer*, **40**, 263.

GOWING, N. F. C. (1970) The Pathology and Natural History of Thyroid Tumours. In *Tumours of the Thyroid Gland.* D. Smithers. Edinburgh and London: E. & S. Livingstone.

GIBSON, R., GRAHAM, S., LILIENFELD, A., SCHUMAN, L., DOWD, J. E. & LEVIN, M. L. (1972) Irradiation in the Epidemiology of Leukemia among Adults. *J. natn. Cancer Inst.*, **48**, 301.

JELIFFE, A. M. & JONES, K. M. (1960) Leukaemia after $I^{131}$ Therapy for Thyroid Cancer. *Clin. Radiol.*, **11**, 134.

LEWALLEN, C. G. & GODWIN, J. T. (1963) Acute Myelogenous Leukemia Complicating Radioactive Iodine Therapy of Thyroid Cancer. *Am. J. Roentg.*, **89**, 610.

Ozarda, A., Ergin, U. & Bender, M. A. (1961) Chronic Myelogenous Leukemia following $I^{131}$ Therapy for Metastatic Thyroid Carcinoma. *Am. J. Roentg.*, **85**, 914.

Pochin, E. E. (1961) The Occurrence of Leukaemia following Radioiodine Therapy. In *Advances in Thyroid Research.* R. Pitt-Rivers. Oxford, London, New York, Paris: Pergamon Press.

Pochin, E. E. (1969) Long-term hazards of Radioiodine Treatment of Thyroid Carcinoma. In *Thyroid Cancer.* C. E. Hedinger. Berlin, Heidelberg, New York: Springer-Verlag.

Saenger, E. L., Thoma, G. E. & Tompkins, E. A. (1968) Incidence of Leukaemia Following Treatment of Hyperthyroidism. *J. Am. med. Ass.*, **205**, 147.

Seidlin, S. M., Siegel, E., Melamed, S. & Yalow, A. A. (1955) Occurrence of Myeloid Leukemia in Patients with Metastatic Thyroid Carcinoma Following Prolonged Massive Radioiodine Therapy. *Bull. N.Y. Acad. Med.*, **31**, 410.

United Nations Scientific Committee on the Effects of Atomic Radiation (1972) *Ionizing Radiation: Levels and Effects.* New York: United Nations.